# Quantifying structural racism in cohort studies to advance prospective evidence

**DOI:** 10.1016/j.ssmph.2023.101417

**Published:** 2023-04-28

**Authors:** Shawna Follis, Khadijah Breathett, Lorena Garcia, Monik Jimenez, Crystal W. Cené, Eric Whitsel, Haley Hedlin, Electra D. Paskett, Shiqi Zhang, Cynthia A. Thomson, Marcia L. Stefanick

**Affiliations:** a Stanford Prevention Research Center, Department of Medicine, Stanford University, 3180 Porter Drive, Mail Code 5702, Palo Alto, CA, 94304, USA; b Division of Cardiovascular Medicine, Indiana University, Indianapolis, IN, USA; c UC Davis School of Medicine, Department of Public Health Sciences, Davis, CA, USA; d Division of Women's Health and Division of Preventive Medicine, Brigham and Women's Hospital, Harvard Medical School, Boston, MA, USA; e University of California, San Diego Department of Medicine, San Diego, CA, USA; f Department of Epidemiology, Gillings School of Global Public Health and Department of Medicine, School of Medicine, University of North Carolina, Chapel Hill, NC, USA; g Quantitative Sciences Unit, Department of Medicine, Stanford University School of Medicine, CA, USA; h College of Medicine, The Ohio State University, Columbus, OH, USA; i Health Promotion Sciences, Mel & Enid Zuckerman College of Public Health, University of Arizona, Tucson, AZ, USA; j Department of Obstetrics & Gynecology and Department of Epidemiology and Population Health, Stanford University, Stanford, CA, USA

**Keywords:** Social determinants of health, Structural racism, Race, Ethnicity, Cohort study, Health, Disparities, Social epidemiology, Office of management and budget

## Abstract

Calls-to-action in health research have described a need to improve research on race, ethnicity, and structural racism. Well-established cohort studies typically lack access to novel structural and social determinants of health (SSDOH) or precise race and ethnicity categorization, contributing to a loss of rigor to conduct informative analyses and a gap in prospective evidence on the role of structural racism in health outcomes. We propose and implement methods that prospective cohort studies can use to begin to rectify this, using the Women's Health Initiative (WHI) cohort as a case study. To do so, we evaluated the quality, precision, and representativeness of race, ethnicity, and SSDOH data compared with the target US population and operationalized methods to quantify structural determinants in cohort studies. Harmonizing racial and ethnic categorization to the current standards set by the Office of Management and Budget improved measurement precision, aligned with published recommendations, disaggregated groups, decreased missing data, and decreased participants reporting “some other race”. Disaggregation revealed sub-group disparities in SSDOH, including a greater proportion of Black-Latina (35.2%) and AIAN-Latina (33.3%) WHI participants with income below the US median compared with White-Latina (42.5%) participants. We found similarities in the racial and ethnic patterning of SSDOH disparities between WHI and US women but less disparity overall in WHI. Despite higher individual-level advantage in WHI, racial disparities in neighborhood resources were similar to the US, reflecting structural racism. Median neighborhood income was comparable between Black WHI ($39,000) and US ($34,700) women. WHI SSDOH-associated outcomes may be generalizable on the basis of comparing across race and ethnicity but may quantitatively (but not qualitatively) underestimate US effect sizes. This paper takes steps towards data justice by implementing methods to make visible hidden health disparity groups and operationalizing structural-level determinants in prospective cohort studies, a first step to establishing causality in health disparities research.

## Highlights

• Leveraging structural racism measures with cohort data would advance prospective evidence on health disparities.
• Harmonizing WHI racial and ethnic categories to the US Census improved precision through disaggregation and standardization.
• Patterning of racial and ethnic disparities in structural and social determinants of health in WHI were similar to the US.

## 1 Introduction

Recent calls-to-action for data justice in health research have emphasized an urgent need to carefully conceptualize race, ethnicity, social determinants of health (SDOH), and structural racism, because the failure to do so perpetuates the hegemonic misinterpretation of health disparities arising from biology or genetics (Adkins-Jackson et al., 2021; Bailey et al., 2017; Boyd et al., 2020; Breathett et al., 2021; Churchwell et al., 2020; Flanagin et al., 2021). Structural racism is a fundamental cause of health disparities, defined as the totality of ways in which multiple systems and institutions interact to assert racist policies, practices, and beliefs about racialized groups (Bailey et al., 2017; Bonilla-silva, 1997). When conceptualized carefully, race and ethnicity remain the central criteria in social stratification and serve as proxies for the unmeasured impact of structural racism on health (Bonilla-silva, 1997; L. Garcia et al., 2022). Theory-driven advances in the conceptual understanding of structural and SDOH (SSDOH) as a fundamental cause of disease justify a hierarchical categorization, with structural-level determinants (i.e., structural racism, residential segregation) creating advantage/disadvantage in access to intermediary-level determinants (i.e., educational attainment) (Churchwell et al., 2020; Powell-Wiley et al., 2022). To operationalize these advances, several calls to action have described methods for effective measurement of race and ethnicity (Breathett et al., 2021; Flanagin et al., 2021) and quantification of interacting forms of structural and intermediary-level determinants (Adkins-Jackson et al., 2021; Boyd et al., 2020). Yet, 90% of research on racism in 2020 still contained no measure of structural racism (Krieger et al., 2021). The difficulty in measuring structural determinants and in disaggregating race and ethnicity contributes to this research gap. Cross-sectional and ecological study designs largely limited to surrogate disease outcomes have efficiently overcome these difficulties, and there is now a need to improve the quality of evidence to analytic and prospective studies (Adkins-Jackson et al., 2021). While prospective cohort studies offer multidimensional risk exposure and adjudicated outcome data to advance research, a critical barrier to data justice is insufficient conceptualization of race, ethnicity, and SSDOH, particularly among cohorts designed prior to the Healthy People SDOH prioritization (Hardeman et al., 2022; King et al., 2020).

The Women's Health Initiative (WHI) is one of the largest and most diverse prospective cohorts of US women ever assembled, with nearly 30 years of longitudinal health data. The WHI is uniquely positioned to implement the methods outlined by aforementioned calls-to-action, with robust life-course data on aging women who have experienced structural racism from historical Jim Crow segregation to residential segregation today, coupled with the intersectional experiences of sexism and ageism. In fact, the WHI was launched to shift research towards greater diversity in response to NIH policy for the equitable inclusion of women and historically marginalized race and ethnicity groups in medical research (later the NIH Revitalization Act) (Institute of Medicine, 1994; The WHI Study Group, 1998\). A recent paper called for the Revitalization Act to require health datasets explain and justify conceptualization of racialized groups (Krieger et al., 2021). While the WHI has contributed important insights into the prevention of cardiovascular disease, cancer, and fractures among postmenopausal women with over 2000 published papers these data have, to date, been under-utilized for the purpose of identifying racial health disparities (LaMonte et al., 2022). The gap in SSDOH and structural racism data across many prospective cohort studies is a barrier to data justice, because it exasperates the paucity of research investigating structural racism (not race) as a fundamental cause of health disparities (Adkins-Jackson et al., 2021; Boyd et al., 2020; Krieger et al., 2021).

This article highlights how ongoing cohort studies can operationalize the methods for data justice described in calls-to-action and Critical Race Theory (CRT) to improve research on race and ethnicity, using key SSDOH constructs and empirical evidence from WHI as an example (Bonilla-silva, 1997; Ford & Airhihenbuwa, 2010). To do so, we followed the Public Health Critical Race (PHCR) Praxis to conceptualize and measure contemporary patterns of racial relationships as sociopolitically patterned by structural racism (Ford & Airhihenbuwa, 2010). The first step, according to Biden's Executive Order (13985) to advance racial equity in the United States (US), is correcting datasets to disaggregate race, ethnicity, and other key demographic variables appropriately (Advancing Racial Equity, 2021). We reconceptualized and harmonized race and ethnicity data to the 2000 US Census Race and Ethnicity Standards (Office of Management and Budget, OMB, 1997) which align more closely with recent recommendations (Flanagin et al., 2021; Ford & Harawa, 2010) and have evaluated the quality and precision of these data in relation to SSDOH in the US target population (L. Garcia et al., 2022). This evaluation provides timely evidence in light of the *Initial Proposals For Updating OMB's R*ace a*nd Ethnicity Statistical Standards (2023)**.* To address the structural data gap in longstanding cohort studies, we highlighted a promising approach to retrospectively quantify structural racism in neighborhood resources using administrative datasets, such as the US Census (Hardeman et al., 2022). Operationalizing high quality and longitudinal data in prospective cohort studies may enable researchers to identify understudied sub-groups and evaluate the causality of SSDOH in racial and ethnic health disparities.

## 2 Methods

### 2.1 Data source and research population

The WHI cohort enrolled 161,808 postmenopausal women aged 50–79 years at baseline (1993–1998) to participate in either an observational study or one or more clinical trials (low-fat diet, menopausal hormone therapy, and/or vitamin D and calcium supplementation) (Hays et al., 2003; The WHI Study Group, 1998). Mass mailings were the primary method used to recruit women at 40 clinical centers throughout the US. The WHI study design and recruitment have been described previously, including recruitment strategies aimed at ensuring that race and ethnicity groups underrepresented in research would represent at least 20% of the cohort (L. Garcia et al., 2022; Larkey et al., 2002). The institutional review board at each clinical site approved the study.

### 2.2 Race and ethnicity data

Race and ethnicity data were first collected during baseline screening for study enrollment (1993–1998) using a self-reported questionnaire that asked participants to select one of six combined-*race/ethnicity* categories: American Indian or Alaska Native; Asian or Pacific Islander; Black or African-American; Hispanic/Latino; White; “other”. New standards have been developed for scientifically accurate conceptualization in biomedical research of race and ethnicity as social not biological constructs (Boyd et al., 2020). While racialization evokes phenotype and ethnicity evokes culture, both are hierarchically indexed through white supremacy creating advantages/disadvantages across SSDOH (Ford & Harawa, 2010). To correct the race and ethnicity measurement in WHI, we use a revised race and ethnicity dataset (L. Garcia et al., 2022) that was operationalized using a second questionnaire administered to active participants in 2003–2004 that categorized race and ethnicity harmonized to the 2000 US Census (OMB Race and Ethnicity Standards 1997 revision), which align more closely with recent recommendations (Breathett et al., 2021; Flanagin et al., 2021; Ford & Harawa, 2010). Because high levels of missing data (N = 24,076) limited the use of the revised questionnaire, in 2021 the WHI imputed the missing data using participant responses from the baseline questionnaire (L. Garcia et al., 2022) Acknowledging the settler colonialism of Latin America by the Spanish/Hispanic, we recommend and use the term “Latina” to refer to ethnicity in place of the OMB term, “Spanish/Hispanic/Latina”. This revised dataset disaggregates Asian, and Native Hawaiian or Pacific Islander (NHPI) races by several nationalities, facilitating research into important sub-group differences (Ford & Harawa, 2010). We present ethnicity and race as separate concepts, so that ethnicity groups encompass any race and vice versa. Participants who selected multiple races were categorized as “two or more races” to maintain comparability with OMB categorization.

### 2.3 Structural and social determinants of health data

The SSDOH and demographic data were collected using self-reported questionnaires at baseline (Matthews et al., 1997). Demographic variables include age and US region of residence. The WHI collected several intermediary-level determinants, including family income, self and partner occupation, health insurance, living with children or relatives, social support, and caregiving. To reflect meaningful credential thresholds with implications for social class, we dichotomized education at bachelor's degree or more (Krieger et al., 1997). Similarly, income was collected as a categorical variable, and we dichotomized it as the proportion reporting an income greater than the US household median income (greater than $34,999 was the closest income category boundary in the WHI and corresponds to 51% of the US (Money *Income in the United States: 1995 and 1996*, 1997)) in 1995 consistent with WHI baseline. Social support, defined as the perception of support from other people, was measured using the validated Medical Outcomes Study short form survey and dichotomized as scores below the median (36 out of 100) indicating low social support (Ware Jr. & Sherbourne, 1992). Caregiving was measured using the Cardiovascular Health Survey (Brown et al., 1990). We describe the SSDOH variables available in WHI in Appendix A.

### 2.4 Structural determinants

A promising approach to operationalize structural determinants and structural racism in cohort studies is the use of geocoding to quantify neighborhood resources (Hardeman et al., 2022). Vast disparities in Black, Latino, and AIAN US neighborhoods are constructed through structural racism across multiple policies and practices, such as racist mortgage lending (John R. Logan, 2011; Kershaw et al., 2015). Neighborhood (census-tract) socioeconomic characteristics were previously geocoded from the 2000 US Census and assigned to WHI participants based on their address of residence (Whitsel et al., 2006). These data provide the WHI with structural-level determinants, and we stratified by race and ethnicity as proxy measures of structural racism. A summary neighborhood socioeconomic status (NSES) Z score based on previously published methods, estimated each participant's neighborhood socioeconomic environment using six variables representing dimensions of wealth, education, and occupation (Diez Roux et al., 2001). Each variable was Z-transformed and summed to construct a summary NSES Z score for each participant. The sum reflects the total deviation from the mean, higher values of which indicate more advantaged neighborhoods. Neighborhood income was measured as the median household income in the participant's census-tract of residence. Neighborhood housing is an indicator of neighborhood wealth and was measured as the median value of housing units. Neighborhood education was measured as the proportion of adults ≥25 years of age in the neighborhood who had completed a bachelor's degree or higher.

### 2.5 Statistical analysis

To describe the WHI cohort characteristics, we compared the disaggregated race and ethnicity variables from the revised dataset by sample size, age, income, and education. The data harmonization to the 2000 US Census race and ethnicity categorization facilitates comparisons to the target population of US women aged 50–79 years using US administrative datasets. We calculated cross-tabulations of Latina ethnicity by racial groups to compare WHI to US women using the standardized categories first available in the 2000 US Census.(Census, 2000) Population pyramids were used to explain the age structure stratified by race and ethnicity between WHI at baseline and US women in 1995 (approximates baseline) using estimates obtained from the US Bureau of the Census Current Population Survey (Day, Jennifer Cheeseman, 1996). Where WHI was compared to the 1995 Census estimates, we maintained comparability by combining WHI Asian and NHPI races (Asian/PI).

We compared ethnic (Latina vs. non-Latina) and racial groups in relation to SSDOH variables across WHI using absolute standardized differences (ASD), a measure of the differences between categories expressed in units of standard deviations (Austin, 2009). Cohen's guidelines were used to interpret the magnitude of the between-group differences; an ASD <0.2 is considered a trivial difference while those of 0.2, 0.5, and 0.8 indicate small, moderate, and large differences, respectively (Cohen, 1988). We compared individual-level income and education self-reported at baseline in WHI to the US population across race and ethnicity using data obtained from the US Bureau of the Census Current Population Survey in 1995 (Day, 1996). Income was compared as the proportion of each race and ethnicity group with income greater than $34,999, approximating the 1995 US median household income (Money *Income in the United States: 1995 and 1996*, 1997). Educational attainment was defined as the race and ethnicity specific proportions of women aged 50–79 years in 1995 with a bachelor's degree or greater (Educational Attainment US, 1995).

Structural-level determinants (neighborhood socioeconomic characteristics) were compared using the ASD between race and ethnicity groups in WHI and compared to the target population where available using the US Census, 2000, consistent with the year that WHI neighborhood data was first available. The WHI neighborhood-level income levels stratified by race and ethnicity were compared with the US population using estimates of census-tract composition of households at the 50th percentile of the year 2000 national income distribution ($42,148) (Reardon et al., 2015). This is interpreted as, the average Latina household in the US lived in neighborhoods where the median income was $36,501 (Fig. 2B). Statistical analyses were performed using SAS, version 9.4 (SAS Institute, Inc., Cary, NC).

## 3 Results

### 3.1 Revised categorization

Table 1 displays the revised race and ethnicity composition of the WHI cohort harmonized to OMB standards and aligned with published recommendations (Adkins-Jackson et al., 2021; Boyd et al., 2020; Breathett et al., 2021; Flanagin et al., 2021; Ford & Harawa, 2010). Measuring ethnicity separately from race in the revised dataset resulted in capturing a greater proportion (4.5%) of the Latina participants compared with the original dataset, in which the rate was 4.1% Hispanic/Latino (original dataset not shown). The revised dataset captured more White participants (85.1% compared with 82.7% in the original dataset), of which many were previously categorized only by their Hispanic/Latino ethnicity. It captured those identifying as multiple races (1.2%), the greatest proportion of whom were American Indian or Alaska Native race (AIAN) (nearly two thirds), with the majority also identifying as White race compared with 40% of AIAN women in the Census 2000. NHPI race (0.1%) is now disaggregated from Asian (2.5%). NHPI race is composed of Native Hawaiian, Guamanian or Chamorro, and Samoan. Asian race in WHI includes 1.5% of participants identifying as Japanese, with Asian Indian, Chinese, Korean, Filipino, and Vietnamese each composing less than 1% of participants. Racial composition was disaggregated by the Latina ethnic group in Table 2 and demonstrated important sub-group differences, with 10.1% of Puerto Rican participants and 0.4% of Mexican, Mexican American, Chicano participants identifying as Black race.

**Table 1:** Baseline characteristics of WHI participants categorized by ethnicity and by race, N = 161,808.

	Sample size N (%)^a^	Age mean ± SD	Income greater than median N (%)^b^	Bachelor's degree or greater N (%)^c^
**Ethnicity** (includes any race)				
**Not Spanish/Hispanic/Latina**	153117 (94.6%)	63.4 (7.22)	85670 (59.8%)	61398 (40.1%)
**Spanish/Hispanic/Latina***(stratified below)*^e^	7312 (4.5%)	60.5 (6.92)	2551 (39.2%)	1587 (21.7%)
*Puerto Rican*	779 (0.5%)	60.7 (6.70)	354 (49.4%)	237 (30.4%)
*Mexican, Mexican American, Chicano*	2693 (1.7%)	59.8 (6.65)	970 (39.6%)	470 (17.5%)
*Cuban*	396 (0.2%)	61.9 (6.57)	137 (39.7%)	148 (37.4%)
*Other Spanish/Hispanic/Latina*	3444 (21%)	60.9 (7.16)	1090 (36.4%)	732 (21.3%)
**Missing**	1379 (0.9%)	63.9 (8.08)	612 (48.9%)	430 (31.2%)
**Race** (by any ethnicity and Spanish/Hispanic/Latina ethnicity only)				
**White**	137628 (85.1%)	63.5 (7.20)	78049 (60.6%)	55191 (40.1%)
Spanish/Hispanic/Latina ethnicity^f^	4300 (58.8%)	60.7 (6.85)	1647 (42.5%)	1002 (23.3%)
**Black or African American**	14327 (8.9%)	61.6 (7.12)	5935 (44.9%)	4903 (34.2%)
Spanish/Hispanic/Latina ethnicity^f^	160 (2.2%)	61.9 (7.36)	51 (35.2%)	51 (31.9%)
**American Indian or Alaskan Native**	540 (0.3%)	61.4 (7.57)	194 (39.4%)	125 (23.1%)
Spanish/Hispanic/Latina ethnicity^f^	53 (0.7%)	59.7 (7.01)	15 (33.3%)	<10^d^
**Asian***(stratified below)*^e^	4025 (2.5%)	63.2 (7.51)	2601 (69.1%)	1832 (45.5%)
Spanish/Hispanic/Latina ethnicity^f^	60 (0.8%)	62.2 (5.93)	28 (50.0%)	22 (36.7%)
*Asian Indian*	83 (0.1%)	58.1 (5.28)	60 (75.9%)	58 (69.9%)
*Chinese*	747 (0.5%)	62.3 (7.01)	537 (78.1%)	447 (59.8%)
*Filipino*	321 (0.2%)	61.4 (6.73)	204 (65.2%)	194 (60.4%)
*Japanese*	1962 (1.2%)	63.9 (7.43)	1306 (70.5%)	772 (39.3%)
*Korean*	91 (0.1%)	63.7 (7.10)	58 (69.0%)	52 (57.1%)
*Vietnamese*	10 (0.0%)	58.5 (5.17)	<10	<10
*Other Asian*	811 (0.5%)	63.8 (8.26)	430 (58.1%)	304 (37.5%)
**Native Hawaiian or Pacific Islander**^e^	137 (0.1%)	60.3 (6.89)	73 (55.7%)	27 (19.7%)
Spanish/Hispanic/Latina ethnicity^f^	18 (0.2%)	58.9 (7.15)	<10	<10
*Native Hawaiian*	97 (0.1%)	60.3 (6.85)	55 (59.1%)	15 (15.5%)
*Guamanian or Chamorro*	10 (0.0%)	56.9 (5.26)	<10 (70.0%)	<10
*Samoan*	<10	<10	<10	<10
*Other Pacific Islander*	28 (0.0%)	60.7 (7.16)	10 (38.5%)	<10
**Two or more races**	1880 (1.2%)	61.8 (7.19)	942 (53.8%)	630 (33.5%)
**“Some other race”**	925 (0.6%)	60.0 (6.98)	373 (44.3%)	254 (27.5%)
**Missing**	2346 (1.4%)	61.3 (7.42)	666 (33.0%)	453 (19.3%)

a Column percent indicates the proportion of all WHI participants who selected the corresponding row ethnicity or race.

b Number of participants in corresponding row that reported family income greater than ($34,999) the national median income in 1995.

c Number of participants in corresponding row that reported educational attainment of high school diploma or greater.

d Sample sizes less than 10 are redacted.

e WHI participants grouped into this racial group if they identified in one of the nationalities stratified below.

f Participants grouped into this category if they selected the specified race and selected Spanish/Hispanic/Latina ethnicity.

**Table 2:** Spanish/Hispanic/Latina ethnicity by racial grouping in WHI and the percent of the US Hispanic population reporting each race (first row), N = 161,808.

			Race					
Ethnicity	White	Black	AIAN	Asian	NHPI	Other race	Two or more	Missing
***US Hispanic***	*47.9%*^a^	*2.0%*	*1.2%*	*0.3%*	*0.1%*	*42.2%*	*6.3%*	
WHI Spanish/Hispanic/Latina	4300 (79.7%)	160 (3.0%)	53 (1.0%)	60 (1.1%)	18 (0.3%)	595 (11.0%)	211 (3.9%)	1915
Not Spanish/Hispanic/Latina	133321 (87.1%)	14166 (9.3%)	292 (0.2%)	3216 (2.1%)	119 (0.1%)	328 (0.2%)	1662 (1.1%)	13
Unknown	10	<10^b^	195 (20.3%)	749 (77.9%)	0	<10	<10	418
***WHI Spanish/Hispanic/Latina Stratified***^c^								
Puerto Rican	536 (73.4%)	74 (10.1%)	<10	12 (1.6%)	<10	64 (8.8%)	31 (4.2%)	49
Mexican, Mexican American, Chicano	2127 (85.7%)	<10	22 (0.9%)	<10	<10	258 (10.4%)	55 (2.2%)	211
Cuban	348 (89.7%)	13 (3.4%)	0 (0.0%)	0	0	19 (4.9%)	<10	<10
Other Spanish/Hispanic/Latina	1289 (71.7%)	64 (3.6%)	25 (1.4%)	40 (2.2%)	<10	254 (14.1%)	117 (6.5%)	1647

Abbreviations: AIAN: American Indian or Alaskan Native; NHPI: Native Hawaiian or Pacific Islander.

a Row percentages indicate the proportion of the corresponding row ethnic group identifying as the column race.

b Sample sizes less than 10 are redacted.

c WHI participants who identified as Spanish/Hispanic/Latina (row 2) are stratified below by their sub-group nationality.

### 3.2 Comparisons to the US

**Fig. 1:**
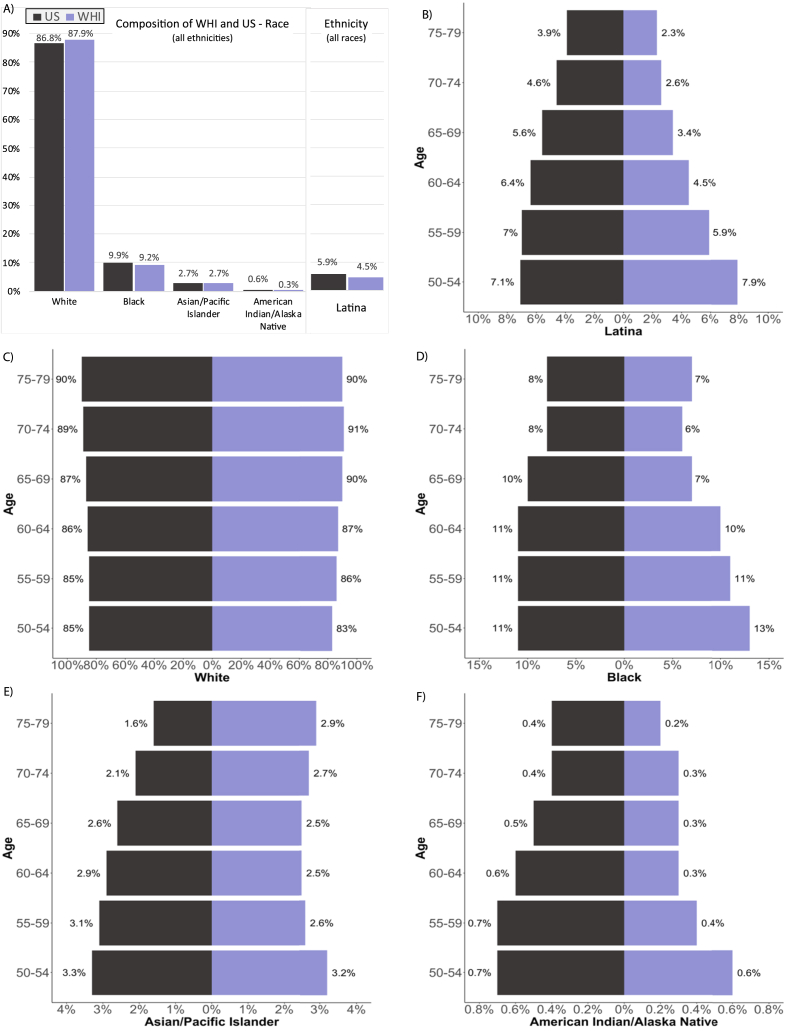
Plot A: the racial and ethnic make-up of US (black bars) compared with WHI (purple bars) women aged 50–79 years in 1995. Plot B: population pyramid comparing ethnicity composition by age between the WHI and US among women aged 50–79 years in 1995. Plots C–F: population pyramid comparing race as described in Plot B. . (For interpretation of the references to color in this figure legend, the reader is referred to the Web version of this article.)

We compared the population proportions of race and ethnicity between the WHI cohort and the target population of US women. Across all age groups a slightly higher absolute percentage of the WHI cohort identified as White race (87.9%), compared with US women in 1995 (86.8%), and equivalent percentages (2.7%) were Asian and NHPI race (Fig. 1A). Overall proportions of Black, Latina, and AIAN women in WHI were underrepresented compared with the US but were representative among the midlife age groups (50–59 years), as displayed by the respective WHI population pyramids in Fig. 1B, D, and 1F. Among participants who reported Latina ethnicity in the WHI, more reported their race as White (79.7%), Asian (1.1%), and NHPI (0.3%) compared with Latina women in the US (47.9%, 0.3%, 0.1%, respectively), and fewer WHI Latina participants (11.0%) selected the “some other race” category compared with 42.2% in the US (Table 2).

**Fig. 2:**
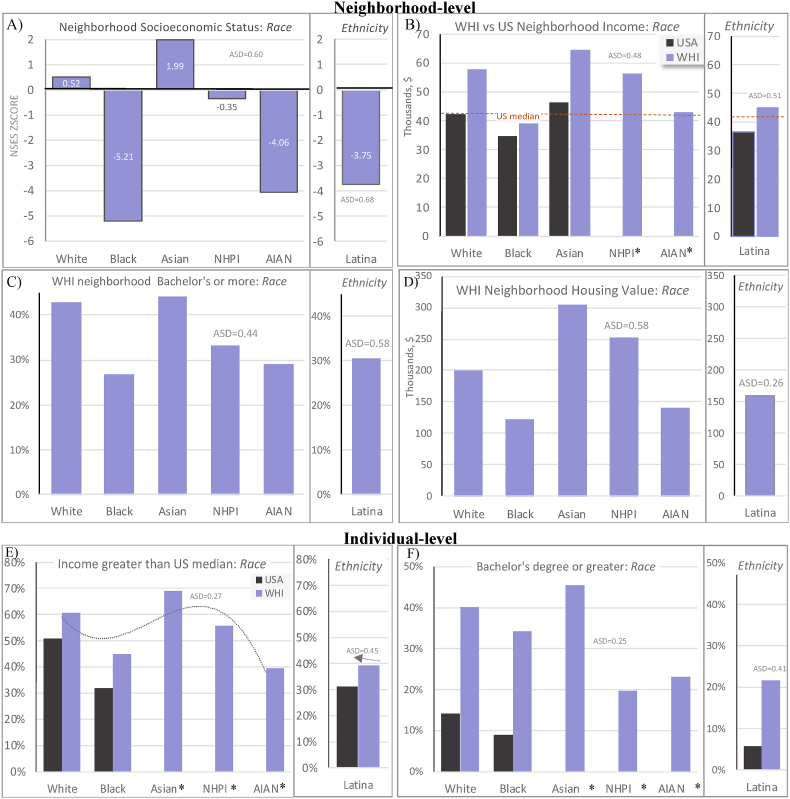
Comparing US Census (black bars) and WHI (purple bars) structural and social determinants of health indicators by race and ethnicity. Plots A–D: structural-level neighborhood socioeconomic characteristics in year 2000. Plots E–F: intermediary-level education and income in year 1995. Absolute standardized differences (ASD) comparing WHI characteristics, first presented across races and then across ethnicities (Latina vs non-Latina). . (For interpretation of the references to color in this figure legend, the reader is referred to the Web version of this article.)

### 3.3 Structural and social determinants of health

Disaggregating key SSDOH variables revealed varying levels of disparities among Black, Latina, and AIAN women across income, education (Table 1), in service and labor occupations, uninsured, caregiving, and social support (Table 3). Disaggregating Latina ethnicity revealed 49.4% of Puerto Rican participants reported income higher than the US median compared with 36.4% of “Other Spanish/Hispanic/Latina” participants, a group including Central Americans, who are the lowest income Latina subgroup in the US (Table 1). Disaggregating Asian participants revealed incomes higher than the US median among 69.1% of Asian and 55.7% of NHPI participants. Comparing the generalizability of SSDOH, the WHI and the US display similar trends across racial and ethnic disparities in individual-level income (Fig. 2E) and education (Fig. 2F).

**Table 3:** Social determinants of health among WHI race and ethnicity groups, N = 161,808.

	Latina ethnicity		Any ethnicity					
	Any race	*ASD*^b^	White	Black	Asian	NHPI	AIAN	*ASD*^b^
Uninsured, n (%)^a^	1395 (19.7%)	0.54	5281 (3.9%)	1186 (8.5%)	97 (2.4%)	<10^c^	56 (10.6%)	0.20
*Missing*	247 (3.4%)		1050 (0.8%)	352 (2.5%)	36 (0.9%)	<10	14 (2.6%)	
Living with children/relatives	2075 (35.8%)	0.45	16040 (11.7%)	3862 (16.9%)	1317 (5.8%)	49 (0.2%)	145 (0.6%)	0.36
*Missing*	1513 (20.7%)		21669 (15.7%)	2688 (18.8%)	343 (8.5%)	<10	106 (19.6%)	
US region of residence		0.70						0.98
Northeast	948 (13.0%)		33455 (24.3%)	2438 (17.0%)	207(5.1%)	<10	57 (10.6%)	
South	2876 (39.3%)		33349 (24.2%)	6847 (47.8%)	254 (6.3%)	<10	143 (26.5%)	
Midwest	318 (4.3%)		31354 (22.8%)	3468 (24.2%)	172 (4.3%)	<10	51 (9.4%)	
West	3170 (43.4%)		39470 (28.7%)	1574 (11.0%)	3392 (84.3%)	119 (86.9%)	289 (53.5%)	
Occupation		0.44						0.28
Managerial/Professional	1725 (26.3%)		53931 (42.6%)	5278 (41.1%)	1661 (42.8%)	46 (34.8%)	153 (31.2%)	
Technical/Sales/Admin	1725 (26.3%)		38242 (30.2%)	3222 (25.1%)	1277 (32.9%)	47 (35.6%)	116 (23.7%)	
Service/Labor	1819 (27.7%)		21088 (16.6%)	3324 (25.9%)	681 (17.6%)	28 (21.2%)	156 (31.8%)	
Homemaker only	1290 (19.7%)		13445 (10.6%)	1019 (7.9%)	259 (6.7%)	11 (8.3%)	65 (13.3%)	
*Missing*	753 (10.3%)		10922 (7.9%)	1484 (10.4%)	147 (3.7%)	<10	50 (9.3%)	
Partner's Occupation		0.38						0.37
Managerial/Professional	1226 (39.9%)		46389 (62.1%)	1825 (39.4%)	1369 (58.3%)	26 (35.6%)	84 (40.6%)	
Technical/Sales/Admin	594 (19.3%)		12415 (16.6%)	584 (12.6%)	438 (18.6%)	13 (17.8%)	31 (15.0%)	
Service/Labor	1188 (38.7%)		15672 (21.0%)	2182 (47.1%)	530 (22.6%)	34 (46.6%)	90 (43.5%)	
Homemaker only	64 (2.1%)		210 (0.3%)	41 (0.9%)	12 (0.5%)	<10	<10	
*Missing*	4240 (58.0%)		62942 (45.7%)	9695 (67.7%)	1676 (41.6%)	64 (46.7%)	333 (61.7%)	
Caregiving	3105 (43.3%)	0.12	55166 (40.3%)	6516 (46.2%)	1245 (31.2%)	41 (29.9%)	251 (47.1%)	0.21
*Missing*	143 (2.0%)		851 (0.6%)	212 (1.5%)	30 (0.7%)	0	<10	
Low social support (score*<36)*	3383 (49.4%)	0.27	52831 (39.3%)	6332 (46.5%)	1614 (41.2%)	58 (43.6%)	255 (50.2%)	0.14
*Missing*	463 (6.3%)		3111 (2.3%)	698 (4.9%)	104 (2.6%)	<10	32 (5.9%)	

Abbreviations: ASD: Absolute standardized differences; AIAN: American Indian or Alaskan Native; NHPI: Native Hawaiian or Pacific Islander.

a Percentage in each row corresponding to a response option is the percent out of the number of people who responded to the question, and the missing percentage is the number of people missing a response divided by the column total.

b ASD comparing WHI characteristics separately first presented across ethnicity (Latina vs non-Latina) and second across races.

c Sample sizes less than 10 are redacted.

A greater proportion of structurally marginalized WHI (Black, AIAN, and Latina) women reported incomes below the overall US median ($35,000) in 1995, while the majority of White, Asian, and NHPI women reported incomes greater than the US median (Fig. 2E). While the Census did not provide full cross-tabulations due to small sample sizes, combined Asian/NHPI income was 16% greater than the 1995 US median, the highest of all races and consistent with the WHI. Latina, Black, and AIAN women on average lived in neighborhoods with NSES levels more than three standard deviations below the WHI population mean (Fig. 2A). Median neighborhood income among WHI women was comparable to US levels, among Black women (WHI: $39,000 versus US: $34,700) in particular (Fig. 2B).

## 4 Discussion

The central role of structural racism in morbidity and mortality has long circulated through health scholarship, CRT, and PHCR (Ford & Airhihenbuwa, 2010; Robinson, 1983) but only recently reached mainstream acknowledgement in response to Black Lives Matter activism (Boyd et al., 2020). We implemented methods recommended for data justice in a longstanding prospective cohort study, resulting in greater precision in reporting of WHI participant's race and ethnicity. Data harmonization to OMB standards improved generalizability across the literature (Breathett et al., 2021; Flanagin et al., 2021; L. Garcia et al., 2022). We implemented methods to address the structural racism data gap using measures of the racialized neighborhood context that align with theoretical frameworks to explain racial clustering of intermediary determinants and health disparities (Adkins-Jackson et al., 2021; Hardeman et al., 2022; Powell-Wiley et al., 2022). WHI women had less disparity across SSDOH than US target population. However, the patterning of racial and ethnic disparities in SSDOH was similar between the WHI and the US. This provides evidence that WHI analytic results comparing SSDOH between WHI race and ethnicity groups (e.g., neighborhood income; NSES which is normalized to the WHI population mean) are generalizable to US patterns.

### 4.1 Revised race and ethnicity composition

The revised WHI race and ethnicity dataset aligns with recommendations to disaggregate race and ethnicity and standardize across datasets, thereby allowing evaluation of generalizability of findings. This approach improved measurement precision compared with the baseline dataset, decreasing missing data overall and the proportion of participants reporting “some other race”. The use of conceptual frameworks, including PHCR, improved the quality and scientific justification of the revised dataset, but race and ethnicity measurement in most cohort studies remains limited to researcher-defined categories attempting to quantify contextually shifting, internally heterogeneous, and intersecting social constructs (Garcia, 2013). In support of recommendations from the *Initial Proposals For Updating OMB's* Race *and Ethnicity Statistical Standards (2023)*, disaggregating race and ethnicity groups in WHI advanced data justice by collecting more granular information that revealed previously hidden marginalized groups. Indeed, disaggregating Asian/NHPI and Latina groups revealed important SSDOH sub-groups differences, while SSDOH disparities remained systemically greater among Indigenous, Black, and Latina compared with White, NHPI, and Asian groups.

The proportion of WHI participants reporting “some other race” decreased by 50% after switching from a 5-category race/ethnicity-combined question to the OMB standards that disaggregate ethnicity from race. Race measurement methods for the Latina ethnicity are particularly vulnerable to methodological effects and have the greatest non-response rate. Indeed, in the 2000 Census 42% of Latino-ethnicity respondents selected “some other race”, likewise, the majority of the “some other race” respondents in WHI reported Latina ethnicity (Census, 2000). In contrast to the Initial Proposals (2023)for Updating OMB, our findings suggest that the recommendation to combine the race and ethnicity question may reduce specificity, concealing the most marginalized racial groups (Black and Indigenous) within the Latino ethnicity. Disaggregating Latina ethnicity revealed sub-group SSDOH disparities and enables future research incorporating CRT evidence on structural racism and pigmentocracy impacting Black-Latina and AIAN-Latina groups (Bonilla-Silva, 2004). For example, there is a need to understand whether the income disparity we identified among Black-Latina compared with White-Latina and Black non-Latina groups is associated with Black-Latina health disparities. We underscore the Initial Proposals (2023) for Updating OMB recommendation for disaggregating the Black race to identify descendants of enslaved Americans and meaningfully classify Africa, as the most genetically and linguistically (ethnically) diverse continent.

Each study must evaluate the generalizability of their findings to the target population, and we provided descriptions to inform extrapolations of WHI race and ethnicity findings. Overall, the race and ethnicity groups in WHI are demographically similar to age-matched US women in 1995. Compared to the US women 50–79 years in 1995, the overall representation and the mean age of WHI Black, Latina, and AIAN women was slightly lower than that of WHI White and Asian/NHPI women. This reflects the strategy to recruit more midlife women from underrepresented groups by halting enrollment of midlife White women when the prespecified proportion was enrolled. Age differences underscore the importance of age correction and stratification commonly practiced in WHI analyses. In WHI most Latina women identified as White race, and most women reporting AIAN race also reported White race; nearly double that of the target population, which may underrepresent US health disparities among non-White AIAN and Latina groups (Humes et al., 2011). Harmonizing the revised dataset to administrative data and other cohorts improves generalizability across the health disparities literature and enables future linkage.

### 4.2 Structural and social determinants of health data

A critical barrier to progress in eliminating health disparities is insufficient data on structural-level SSDOH data in longitudinal cohort studies with rich exposure and outcome data (Adkins-Jackson et al., 2021; Boyd et al., 2020). Despite some limitations in terms of the totality of variables used to described intermediary-level SSDOH in the literature (e.g., acculturation and discrimination) the WHI dataset includes all Healthy People defined SSDOH domains (Appendix A), thus providing a rich descriptive base from which meaningful interpretation can be made to inform on racial and ethnic health disparities. Social support and caregiving represent several measures of the “social and community context” domain (Follis et al., 2021). Social class and material resources can be indicated by occupation, income, and education variables. Partner's education and occupation reflect interpersonal-level SSDOH and may provide greater accuracy among older age women (Grundy & Holt, 2001). The etiology of social risk can be investigated with SSDOH capturing timing and duration across the life-course, including region of birth, age at first job, and immigration, an important effect modifier in Latina health disparities (the Hispanic Paradox).

Comparing the generalizability of WHI race and ethnicity groups in relation to SSDOH suggests consistently lower levels of disadvantage in WHI in relation to the target US population; however, the patterning of SSDOH disparities among WHI race and ethnicity groups are representative of structural racism in the US (Bonilla-silva, 1997). For example, while income is higher among all WHI women compared with the US, the greatest disparities in both populations are among Black, AIAN, and Latina women and greatest advantage among Asian women. This supports the generalizability of research findings on SSDOH disparities between WHI race and ethnicity groups, though researchers should consider whether findings quantitatively (but not qualitatively) underestimate effect sizes in the target population. The opportunity to investigate race-specific positive health effects of educational attainment in WHI would be well powered, with 45% of Black women having college degrees. Vast differences in SSDOH between race, ethnicity, and nationality groups highlight the need for future research to disaggregate health outcomes among these groups, and the imprecision of aggregating separate races and ethnicities into an “other races” category without evidenced justification.

Neighborhood socioeconomic characteristics offer many advantages providing WHI with structural-level measures and greater explanatory power for health disparities among older adults when income after retirement loses social class significance (Grundy & Holt, 2001). Overall WHI patterns parallel wider US population patterns in structural racism, with systematically higher advantage on average among WHI participants. Despite higher individual-level advantage in WHI, there were some similarities with US racial disparities in neighborhood resources, reflecting structural racism. Median neighborhood income was comparable among Black women (WHI: $39,000 and US: $34,700). Research aligns with theory to show the primacy of racialization in contextual social organization with more *affluent* Black and Latino individuals living in resource-deprived neighborhoods than do *poor* White individuals (John R. Logan, 2011; Kershaw et al., 2015). CRT has demonstrated that higher individual-level social class does not prevent structural racism but are intrinsically tied, coined racial capitalism (Bonilla-silva, 1997; Robinson, 1983). These findings support the generalizability of future WHI research investigating the role of race and ethnicity specific neighborhood environments in health disparities. Extending WHI Census-derived measures to create recommended structural racism indices is underway, including racial residential segregation, as demonstrated in the Coronary Artery Risk Development in Young Adults Study (Adkins-Jackson et al., 2021; Hardeman et al., 2022; Kershaw et al., 2015). These data in WHI would permit statistically powered investigation of race and ethnicity groups, causal inference, and multilevel SSDOH, which is a research priority (Churchwell et al., 2020).

### 4.3 Recommendations and limitations

This paper has many strengths, including a large sample size of race and ethnicity groups and several intermediary-level SSDOH available in WHI. Consideration of multilevel SSDOH is crucial to analysis, interpretation, and generalizability of racial and ethnic health disparities to the target population, and our results provide a framework for investigators to do so (Churchwell et al., 2020). Our findings suggest analytic results for the effects of SSDOH on health outcomes in WHI may systematically underestimate US effects and disproportionately underestimate effects among US health disparity populations, a common limitation across many cohorts impeding anti-racist health policy (Hardeman et al., 2022). Likewise, WHI and many longstanding cohorts lack direct measures of structural racism (Krieger et al., 2021). New data harmonization between administrative datasets and the revised WHI race and ethnicity dataset now facilitates methods to create structural racism measures from administrative datasets and statistical techniques to achieve better generalizability to the target population. The latter recommendation uses weighting techniques to calibrate populations levels of sociodemographic and SSDOH across the revised race and ethnicity groups in WHI to that of the US target population from administrative datasets. For example, post-stratification weighting using external race and ethnicity data from the US Census (e.g., Fig. 2B) can be used to correct the systematic underrepresentation in WHI of the most marginalized from the target population, with the capability of correcting for selection-bias. Linking administrative data can overcome limitations of self-reported income data, such as nonresponse bias, and self-report bias.

Disaggregation of race and ethnicity groups in longstanding cohort studies with large sample sizes of clinical events (e.g., 3161 CVD events among Black WHI participants) can be sufficiently powered for stratified statistical analyses. However, the small sample size for several race and ethnicity groups is a limitation for statistical powered analyses. The common practice of aggregating small sample size categories into “other” is unscientific. One analytic solution is to present descriptive analyses of sub-groups underpowered for the main analysis (Ward et al., 2019). Sufficiently powered analyses may require cautious data aggregation using research question specific, theory-informed decision making. For example, our results reflect some commonalities in forms of structural racism experienced by Black and AIAN groups within both the Latina and non-Latina ethnicity (Bonilla-Silva, 2004). Racial capitalism is one form that similarly increased COVID-19 risk among Black, Latino, and AIAN communities (Laster Pirtle, 2020). Indeed, CRT scholars posit similar positions on the hierarchy of structural racism are anchored to a pigmentocratic racial system, providing theory and evidence to test hypotheses for aggregating groups experiencing racial capitalism (Bonilla-Silva, 2004). Conceptualization of contemporary patterns of racial relationships as socio-politically patterned by structural racism should be guided using frameworks, such as PHCR (Ford & Airhihenbuwa, 2010). Categorization of the growing multiracial population should be based on self-identification, and any attempts at grouping should be justified by the multiple dimensions of racial identify formation theory as a function of historical legacies and socialization (Garcia, 2013). Novel race and ethnicity measurement methods (i.e., perceived race and colorism) grounded in aforementioned theories may reveal dimensions of structural racism beyond the limitations of OMB categories, but future research is needed to evaluate integration into cohort studies (Ford & Harawa, 2010; Garcia, 2013).

### 4.4 Conclusion

We demonstrated data justice methods for longstanding cohort studies to improve research in racial and ethnic health disparities and SSDOH. The revised race and ethnicity dataset enabled data harmonization and improved generalizability across the health disparities literature. Evaluating the change in WHI race and ethnicity categorization informs the ongoing discussion of proposed changes to the OMB standards. Characterizing the extent to which WHI race and ethnicity results are generalizable to the target population in relation to underlying SSDOH has data justice implications to inform effective analyses and reporting of health disparities. We described SSDOH disparities within understudied subgroups to make visible previously hidden health disparities and to call for future research on subgroups. Given the substantial financial and human resources dedicated to the issue of health disparities, implementing these methods in prospective cohort studies advances research on structural racism as a fundamental cause. This is a necessary step towards identifying pathways through which structural determinants impact health disparities. Future research is needed to develop robust and valid measures of structural racism in prospective cohort studies to evaluate causal dimensions of the relationship between structural racism, SSDOH, and health disparities.

## Sources of funding

Dr. Breathett received grant support from National Heart, Lung, and Blood Institute K01HL142848, R56HL159216, R01HL159216, and L30HL148881.

The WHI program is funded by the National Heart, Lung, and Blood Institute, National Institutes of Health, U.S. Department of Health and Human Services through 75N92021D00001, 75N92021D00002, 75N92021D00003, 75N92021D00004, 75N92021D00005.

## Author agreement

All authors certify that we have seen and approved the final version of the manuscript being submitted.

## Author Contributions

All authors significantly contributed to this paper according to the CREDIT author statement, including roles: Conceptualization, Methodology, and Writing – review & editing. Shawna Follis and Shiqi Zhang additionally contributed to the Formal analysis.

## Declaration of interest

Electra Paskett is multiple PI on grants to the institution from Merck Foundation, Pfizer, and Genentech not related to this work. There is no other financial nor personal interest or belief that could affect their objectivity.

## Data Availability

The authors do not have permission to share data.
