# Supplementary material for: Quantifying structural racism in cohort studies to advance prospective evidence
Source: SSM Popul Health. 2023 Apr 28;22:101417. doi: 10.1016/j.ssmph.2023.101417 (PMC10189286; doi:10.1016/j.ssmph.2023.101417)
Supplement: Multimedia component 1 [file mmc1.docx]

**Supplementary Material**

**Table 1.** Several structural and social determinants of health variables available in the WHI, organized by Health People 2030 Social Determinants of Health Domains

| **Domain 1: Economic stability** |
| --- |
| Family income |
| Occupational class |
| Partner’s occupation |
| Age at entry to labor market |
| Barriers to eating |
| Food assistance |
| Finances better 3 years ago |
| Total family savings |
| Total family debt |
| Hard time making ends meet 3 years ago |
| **Domain 2: Education access and quality** |
| Education |
| Partner’s education |
| Language |
| **Domain 3: Health care access and quality** |
| Health care and insurance status |
| Time since last medical visit |
| Vaccination status |
| **Domain 4: Neighborhood & built environment** |
| How many people in household? |
| Do you live with children or relatives? |
| US region of residence |
| Geocoded neighborhood resources |
| **Domain 5: Social and community context** |
| Social support |
| Caregiving |
| Social Functioning |
| Social Strain |
| Social Integration |
| Immigration history |
| Immigration history of parents |
| Brief Resilience Scale |
| Geocoded neighborhood socioeconomic status index |
